# Medical News and Miscellany

**Published:** 1892-01

**Authors:** 


### Medical News and Miscellany.

**Dr. J. D. Wingate** has removed from Llano to Comanche.

**Dr. J. C. Anderson**, of Granger, Texas, is at the St. Louis polyclinic.

**Push Yandell's** proposal to organze a Public Health Association in Texas.

**Dr. Eugene Clark**, of Lockhart, has gone to New York, to attend the polyclinic.

**Dr. C. W. Simpson**, of Ferris, has gone to New York, to attend the post graduate medical college.

**A Happy New Year!** to all the JOURNAL'S readers. MAY THEY ALL "LIVE LONG and prosper!"

**The** JOURNAL regrets to chronicle the death of Dr. F. E. Yoakum's father, recently, at Greenville, the old home.

**A discharged lunatic**, at Chicago, shot and killed Dr. Flavin Wheeler, on whose testimony he had been pronounced insane.

**Dr. H. L. Hillgarten**, oculist, of Austin, Texas, has just returned from a two months' visit to his old home in Baltimore.

**Remember**—The big State Medical Association meeting will take place at Tyler the fourth Tuesday in April, this year. President Wilkes' speech will be a treat.

**Many Members** of the Central Texas Medical Association attended the meeting of the Austin District Association, December 17, and made lots of friends while here.

---

**From Chihuahua**, Mexico: A sand storm prevailed all day December 31st, and at 1 o'clock p. m. lifted the roof off of the splendid large hospital of the M. C. R. R. Company. No lives lost.

---

**Subscriptions** for volume seven, beginning July, 1891, are long past due; an early remittance is respectfully requested. "Betty and the baby" need shoes and stockings to keep the wolf from the door.

---

**Dr. R. Atkinson**, of San Marcos, sustained a sad bereavement recently, in the loss of his mother. THE JOURNAL tenders its sincere condolence. Mrs. Atkinson was a noble woman, one whose practical benevolence will be missed by many of the less fortunate in life.

---

**Dr. Frank S. White**, who has been appointed Superintendent of the State Lunatic Asylum at Austin, is only 32 years of age—the youngest man, we presume, ever to attain a position of such eminence and responsibility. But he has all the qualities to make a success. Success to him.

---

**Dr. Matt Smith**, of Austin, has been elected Resident Physician of the city and county hospital at Austin, vice F. P. McLaughlin. Dr. McLaughlin held the position many years, filling the requirements to the entire satisfaction of the administration. He prefers now to give his entire time to his growing private practice.

---

**Dr. B. E. Hadra** has returned to Galveston, and located permanently. The medical press, throughout this country and in Europe, have very generally copied and commented favorably upon his device of wiring the vertebra for fracture, and for Pott's disease. The *London Lancet*, in recent issue, speaks favorably of the operation.

---

**Errata**.—In our December number, the names of the writers were omitted from the two communications on "screw worms" in the nose. The letters were written, one by Dr. T. C. Osborn, Cleburne, and the other by Dr. T. J. Turpin, Laredo. The names appeared in the index only. We apologize to the gentlemen for this accident.

---

**Dr. E. G. Cochran** has been appointed assistant surgeon in charge of the Mexican Central Railway Hospitals, at Tampico, Mexico, *vice* Dr. C. M. Harrison, who has been compelled to give up the work, on account of his health. Dr. Harrison has removed to Silao, Mexico. *Apropos*, Dr. Cochran was made happy by the advent of a son, his first born, who came in a few days ahead of Santa Claus, arriving on 22nd of December.

---

**Dr. J. C. McKean**, of Corn Hill, Williamson county, lost his residence and entire contents by fire, on the night of 16th Dec. As his office was in his residence, he lost also all his library, instruments and appliances, and what is even a harder blow, lost $400 in cash, all the money he had, he says. The JOURNAL sympathises deeply with the Doctor in his great misfortune. As the season for collections is pretty well over in the country, the loss falls with terrible force on him and his young family.

---

**Dr. Van B. Thornton & Son**, of Hempstead, Texas, are looking around with a view of establishing a hospital at some point for the treatment of diseases of the skin especially; but will receive other cases. The JOURNAL learns that they have had a large experience in treating certain diseases of the skin and mucous membrane, which are usually denominated "cancer" by the laity and by designing empirics, and have been very successful. What city will donate a building site for this institution? Austin and Lampasas have been thought of as affording the best advantages.

---

The *Texas Sanitarian* for December is, if possible, an improvement on its first issue. It has come to stay, it seems. The reception given it by the medical press is most cordial. It strikes a popular key; the better class of non-medical readers, as well as leading physicians, are subscribing for it, and doctors, school teachers, lawyers, etc., speak well of its beginning. Its prospects are certainly flattering. Ably conducted and high toned, it will not stoop to personaities, nor engage in profitless controversies. It deserves, and doubtless will receive, a cordial support. The JOURNAL will club subscriptions with the *Sanitarian* at $3 for the two, the conditions being that the party is not already a subscriber.

Prof. J. E. Thompson, M. D., Surgeon of the Faculty of the Texas Medical College (University of Texas, Medical Department), at the banquet, given at the close of the recent session of the Austin District Medical Society, in response to a toast, said, unlike Martin Chuzzlewit and Mark Tapley on their visit to America, he had not been asked "What do you think of our country?" nor had he been told that "that is one of the most remarkable men this country ever produced" with reference to a single Texan, and he had met some of the celebrities. But that he had learned, as did Martin and Mark, that it is good policy to admire everything shown him in the way of public enterprise; and, indeed, there was much to admire. He was incautious enough one day, he said, to express an opinion in the presence of a lady in Galveston, to the effect that the streets and sidewalks in that city were not quite as good as they have in London or Manchester (the Doctor is an Englishman, and just from Manchester), whereupon the lady flared up and indignantly replied, that they were "a sight better than they had in Houston, anyhow." He says, that in New York city once, in getting down from an elevated railroad, he, like "Captain Foster"—you know, who "went to Gloster," "stepped in a puddle up to his middle,"—and that he "never went there again." Dr. Thompson is not a bit English; he adapts himself readily to the people "and institutions" of Texas, and is a real genial and humorous gentleman; he can see a joke and can tell one—unlike most of his countrymen.

---

A Disgraceful Swindle.—A leading physician in San Antonio sendf the JOURNAL the following shameful document, and says several of his patients there have been victimized by this heartless she fiend. The first document is printed, on a 6x8 slip of newspaper paper; the "recipe"—the *thing* she gives for the five dollars, is written in a schoolboy hand on a piece of dingy white foolscap paper, and is here copied *verbatim:*

"THE LADIES' FRIEND.

"For Five Dollars I will sell a Recipe to prevent Further Increase in Family. No medicine or instruments of any kind to be used. Healthy, easy, and not injurious to the health. This does not stop the increase entirely; you can prolong the time to suit yourself. Neither does it interfere with the relations of husband and wife at any time, nor is it necessary to keep count of days.

"This has baffled the skill of the most eminent physicians for ages, but at last has come to light, and is worth *Five Hundred Dollars* to any one, and I will also give One Hundred Dollars to any one following the rules and it fails to do as represented.

"Parties living at a distance can send me Five Dollars through the mail, and I will send Recipe promptly by return mail. Parties living in the city can drop me a notice through mail, and state just when they wish to see me, and I will call at their residence.

"Anyone that will get up a club of ten will receive one recipe, free, or fifty cents on each and every recipe by any person's influence. Satisfaction Guaranteed or money refunded.

"Address, Mrs. M. H. Lewis, San Antonio, Texas. Office, 19 North Flores St. in Favorite Restaurant. Office hours, 12 until 2 p. m., and from 5 to 9 p. m."

"THE LADIS FRIEND

"Never Catch your breath when thare is eny dainger of concepion alwais hold your breath at least one half minute at the time of your Husbans discharge and the conception will return if you will folow this rule I will garntee you not bare and if at eny time you should fail to hold your breath take a cold bath ameadtly after intercourse. M. H. LEWIS."

The cunning displayed in this trap is only equaled by the heartlessness of the swindle. No harm can result from following the "directions" (unless it be from the cold bath at an unseasonable hour),.and that is the only thing that can be said in mitigation of the fraud. In this respect it is not so bad as some other means recommended even through the religious press. It would seem that intelligent people could hardly be taken in on so barefaced a promise; but all are not intelligent; and even amongst those who are, there are many wives who find the cares and expenses of a growing family are wearing their lives away, and without a thought of sin or evil, would, if they could, limit or stop the child bearing. They readily catch at anything offered them, especially by one of their sex, which promises the results without pain, danger, medicine or instruments, and which it is promised will not interfere with the marital rights of the husband. These women are to be pitied, and the "strong arm of the law" should protect them from this she-devil. It is worse than stealing. But—how can the law reach her? It certainly is *swindling*—obtaining money under false pretense; but who will cause the arrest? Judging from the word Galveston printed at the head of the recipe, this woman has been plying her nefarious game in that city. The JOURNAL gives publicity to the affair in the hope that its readers will warn their patients against the deception. Nothing will prevent conception except non-intercourse. Holding the breath is perhaps as harmless as any other effort (?) in that direction.

---

Much evidence is adduced to show that diphtheria has often been conveyed through the medium of milk; and the fact that cows, as well as cats, can be inoculated with the bacillus diphtheria gives strong ground for believing that the infectivity of milk is due to some disease of the cow. Accordingly, the author urges that raw milk should never be used, but that it should always be raised to a temperature of 155° F., and kept at that heat for at least six minutes.

There is one other point to which the author refers that may well be considered, and that is, that teachers often receive convalescents into school at too early a date, and do not exclude children of the same family presenting incipient symptoms of illness, because the financial condition of the school or the teacher's salary is dependent upon the average attendance. This is an evil that has been referred to in this country, and it seems that it would be to the interest of school boards and of health boards to agree upon some definite plan that would not work injustice to either pupil or teacher.—*N. Y. Med. Journal.*

